# The active form of *Helicobacter pylori* vacuolating cytotoxin induces decay‐accelerating factor CD55 in association with intestinal metaplasia in the human gastric mucosa

**DOI:** 10.1002/path.5990

**Published:** 2022-08-18

**Authors:** Kazuyo Kaneko, Abed M Zaitoun, Darren P Letley, Joanne L Rhead, Javier Torres, Ian Spendlove, John C Atherton, Karen Robinson

**Affiliations:** ^1^ Nottingham Digestive Diseases Biomedical Research Centre Nottingham University Hospitals NHS Trust and University of Nottingham Nottingham UK; ^2^ Department of Cellular Pathology Nottingham University Hospitals NHS Trust, Queen's Medical Centre Campus Nottingham UK; ^3^ Unidad de Investigación en Enfermedades Infecciosas Hospital de Pediatría, Centro Médico Nacional Siglo XXI, IMSS Mexico City Mexico; ^4^ Division of Cancer and Stem Cells, School of Medicine University of Nottingham Biodiscovery Institute Nottingham UK

**Keywords:** *Helicobacter pylori*, vacuolating cytotoxin, decay‐accelerating factor, CD55, intestinal metaplasia, gastric cancer, virulence factor

## Abstract

High‐level expression of decay‐accelerating factor, CD55, has previously been found in human gastric cancer (GC) and intestinal metaplasia (IM) tissues. Therapeutic effects of CD55 inhibition in cancer have been reported. However, the role of *Helicobacter pylori* infection and virulence factors in the induction of CD55 and its association with histological changes of the human gastric mucosa remain incompletely understood. We hypothesised that CD55 would be increased during infection with more virulent strains of *H. pylori*, and with more marked gastric mucosal pathology. RT‐qPCR and immunohistochemical analyses of gastric biopsy samples from 42 *H. pylori*‐infected and 42 uninfected patients revealed that *CD55* mRNA and protein were significantly higher in the gastric antrum of *H. pylori*‐infected patients, and this was associated with the presence of IM, but not atrophy, or inflammation. Increased gastric CD55 and IM were both linked with colonisation by *vacA* i1‐type strains independently of *cagA* status, and *in vitro* studies using isogenic mutants of *vacA* confirmed the ability of VacA to induce CD55 and sCD55 in gastric epithelial cell lines. siRNA experiments to investigate the function of *H. pylori*‐induced CD55 showed that CD55 knockdown in gastric epithelial cells partially reduced IL‐8 secretion in response to *H. pylori*, but this was not due to modulation of bacterial adhesion or cytotoxicity. Finally, plasma samples taken from the same patients were analysed for the soluble form of CD55 (sCD55) by ELISA. sCD55 levels were not influenced by IM and did not correlate with gastric *CD55* mRNA levels. These results suggest a new link between active *vacA* i1‐type *H. pylori*, IM, and CD55, and identify CD55 as a molecule of potential interest in the management of IM as well as GC treatment. © 2022 The Authors. *The Journal of Pathology* published by John Wiley & Sons Ltd on behalf of The Pathological Society of Great Britain and Ireland.

## Introduction

Chronic infection with *Helicobacter pylori* is the primary cause of gastric cancer (GC), the fifth most common malignancy worldwide and the fourth leading cause of cancer‐related death [1]. Almost 50% of the world's population are colonised from early childhood and throughout life [2]. Persistent *H. pylori* infection may lead to a stepwise histological progression from chronic gastritis through atrophy, intestinal metaplasia (IM), dysplasia, and finally resulting in intestinal‐type gastric adenocarcinoma [3]. IM is characterised by transdifferentiation of gastric epithelial cells to intestine‐specific lineages such as goblet and Paneth cells, and may be defined as complete (type I; well‐developed goblet cells and absorptive columnar cells with a well‐defined brush border) or incomplete (variable sized goblet cells and mucous columnar cells with no brush border), which can be further divided into type II (predominately sialomucins in columnar cells) and type III (predominately sulfomucins in columnar cells) [4]. Histological IM subtyping is not routinely recommended in the current MAPS (management of precancerous conditions and lesions in stomach) guidelines [5] due to poor reproducibility, although incomplete IM has been suggested to confer a greater risk of progression to GC [6]. Patients with extensive IM, affecting both antrum and corpus, are at a greater risk of developing GC and endoscopic surveillance is recommended every 3 years [5, 7]. *H. pylori* eradication can reduce the risk of GC development, but only in patients without IM or dysplasia [8, 9]. Furthermore, widespread *H. pylori* eradication is a concern, particularly in relatively low GC risk regions such as the UK, as it may impact antibiotic resistance and the potential protective benefit of *H. pylori* infection against the development of allergy and autoimmune diseases such as multiple sclerosis [2]. While only 1–5% of infected individuals develop GC, the prognosis is often very poor because the late appearance of symptoms means that the cancer is at an advanced stage when diagnosed. Thus, there is an urgent need for early prognostic biomarkers of GC risk and preventive interventions for patients with precancerous conditions.

The risk of developing *H. pylori*‐mediated GC is related to a complex interplay between host genetics, environmental factors, and bacterial virulence properties [2]. Among several strain‐specific virulence factors identified, the cytotoxin‐associated gene pathogenicity island (*cag*PAI) and vacuolating cytotoxin A (VacA) have been most extensively studied in association with GC. The *cag*PAI gene encodes a type IV secretion system (T4SS) that delivers the effector protein CagA into gastric epithelial cells and stimulates various cell signalling processes [2]. The *cagE* gene is essential for the assembly of a functional T4SS [10]. *In vitro*, CagA signalling causes gastric epithelial cells to develop long projections, a morphological change known as the hummingbird phenotype [11]. *cag*PAI is present in about 70% of *H. pylori* strains [12], and accumulated evidence indicates that infection with a strain possessing a functional *cag*PAI increases the risk of GC and precancerous conditions including IM and dysplasia, compared with *cag*PAI^−^ strains [13, 14, 15, 16].

VacA is a pore‐forming toxin which induces multiple effects in cells *in vitro* [17]. All strains of *H. pylori* possess a *vacA* gene, but VacA‐mediated effects on host cells differ between strains and this is attributed to allelic diversity mainly in the signal (s1/s2), intermediate (i1/i2), and mid (m1/m2) regions. Expression of s1i1‐type VacA induces more vacuolating activity *in vitro* than other forms, and such strains are associated with an increased risk of GC and IM [13, 15, 16, 18]. However, the presence of s1 *vacA* alleles and *cag*PAI are strongly linked [19], making it difficult to define the exact role of VacA in the pathogenesis of GC. We have reported previously that the *vacA* i1 genotype is a more accurate marker of GC risk than other *vacA* alleles or *cagA* status [18]. In a mouse model, infection with *cag*PAI^−^ strains expressing s1i1 forms of VacA caused more severe and extensive metaplasia. Similarly, *vacA* s1i1‐type strains were strongly associated with the presence of IM in the gastric mucosa of patients independent of *cagA* status [20]. How VacA induces IM and GC remains to be elucidated.

CD55, also known as decay‐accelerating factor (DAF), is a glycosylphosphatidylinositol‐anchored membrane protein that protects host cells from autologous complement‐mediated damage. It accelerates the decay of C3 convertases, inhibiting the downstream assembly of the membrane attack complex which functions to eliminate invading pathogens [21]. CD55 also modulates T‐cell responses independent of complement regulation by binding to CD97, expressed on various types of immune cells [22, 23]. A broad range of cancer types overexpress CD55, and its involvement in tumour development and progression has been suggested [21]. Early histological studies of gastric biopsies demonstrated strong CD55 expression in IM, gastric adenoma, and intestinal‐type adenocarcinoma [24, 25]. More recently, *H. pylori* infection of gastric epithelial cell lines and mouse gastric tissues resulted in induced expression of CD55 in a *cag*T4SS‐dependent manner [26, 27]. However, data in humans are limited and inconsistent [24, 25, 28], and the effects of *H. pylori* infection on CD55 in the human stomach remain poorly understood. A soluble form of CD55 (sCD55) has also been detected in blood and other body fluids [29] but, to date, there are no published data on sCD55 in relation to *H. pylori* or gastric disease status.

We hypothesised that CD55 would be increased during infection with more virulent strains of *H. pylori*, and with more marked gastric mucosal pathology. The present study aimed to examine whether membrane‐bound and soluble forms of CD55 are associated with histological changes in the human gastric mucosa induced by different *H. pylori* strain types. We found that increased CD55 expression on *H. pylori*‐infected human gastric mucosa was associated with IM, and this was linked to *vacA* i1‐type strains. Furthermore, *in vitro* studies showed that VacA can induce CD55 and that CD55 is partly involved in *H. pylori*‐mediated IL‐8 secretion in gastric epithelial cells. However, plasma sCD55 levels were not influenced by IM, atrophic gastritis (AG) or *H. pylori* status.

## Materials and methods

### Patients and clinical materials

Gastric biopsy and peripheral blood samples from 84 patients (aged 21–86 years) undergoing a routine upper gastrointestinal endoscopy, most commonly for dyspepsia, at the Queen's Medical Centre, Nottingham, UK between 2003 and 2013, were analysed retrospectively. All patients gave written informed consent with approval of the Nottingham Research Ethics Committee 2 (08/H0408/195). Patients regularly taking high‐dose NSAIDs or antibiotics in the preceding 2 weeks were excluded. For some patients, either a biopsy or a blood sample was not available. *H. pylori* status was determined by serology, rapid urease test, histology, and bacterial culture. *H. pylori* isolates were genotyped for *vacA* and *cagA* by PCR [18, 20]. *H. pylori‐*negative patients were matched for age and gender with infected patients. The characteristics of the patients are shown in Table 1.

**Table 1:** Characteristics of patients in the Nottingham cohort.

	*H. pylori‐*negative (*n* = 42)	*H. pylori‐*positive (*n* = 42)
Gender		
Female, *n* (%)	21 (50.0%)	21 (50.0%)
Male, *n* (%)	21 (50.0%)	21 (50.0%)
Age (years)		
Mean ± SD	59.9 ± 13.8	61.3 ± 13.7
Endoscopic findings		
Normal/healed, *n* (%)	20 (46.5%)	23 (54.8%)
Gastric ulcer/erosion, *n* (%)	11 (25.6%)	14 (33.3%)
Duodenal ulcer/erosion, *n* (%)	12 (27.9%)	6 (14.3%)
Dysplasia, *n* (%)	0 (0%)	0 (0%)
Gastric cancer, *n* (%)	0 (0%)	0 (0%)

### Cell culture, *H. pylori* strains, and culture

AGS (ECACC 89090402) and MKN28 (JCRB 0253) human gastric epithelial cell lines were maintained in RPMI 1640 medium supplemented with 10% FCS, 100 U/ml penicillin, and 100 μg/ml streptomycin (Sigma‐Aldrich, Dorset, UK) at 37 °C in 5% CO_2_. *H. pylori* strain 60190 and its isogenic *cagA*, *cagE*, and *vacA* null mutants, and SS1 mutants expressing s1i1, s2i2 (wild type) or null *vacA* [20, 30] were grown on blood agar plates (Oxoid, Basingstoke, UK) at 37 °C under microaerobic conditions. Bacterial water extracts were prepared as described previously [31]. For *in vitro* experiments, cells were seeded at 2 × 10^5^ per well in 12‐well plates in the absence of antibiotics and treated the following day with bacteria or bacterial water extracts for 24 h in fresh serum‐free medium.

### siRNA transfections

AGS (1.2 × 10^5^ per well) and MKN28 (2 × 10^5^ per well) cells were transfected with 10 nm Silencer Select siRNAs in 12‐well plates using Lipofectamine RNAi MAX reagent (Thermo Fisher Scientific, Waltham, MA, USA). After 48 h, cells were transferred to serum‐free medium and infected with *H. pylori*. Cell viability was determined using a standard MTT [3‐(4,5‐dimethylthiazol‐2‐yl)‐2,5‐diphenyltetrazolium bromide] assay (Sigma‐Aldrich, St Louis, MO, USA) 24 h after *H. pylori* co‐culture. In brief, after supernatants were harvested for ELISA, cells were incubated with fresh medium containing 0.5 mg/ml MTT for 3 h at 37 °C. Subsequently, the medium was removed and 500 μl of 0.04 m hydrochloric acid in isopropanol and 50 μl of 10% SDS were added to each well. The absorbance of solutions was measured at a wavelength of 595 nm. A decrease in absorbance indicates a reduction in the number of viable cells. For bacterial adhesion assays, cells were washed twice with PBS and harvested by scraping in 1 ml of PBS. Ten‐fold serial dilutions of cell suspensions were spotted onto blood agar plates and colonies counted after 3–5 days’ incubation under microaerobic conditions.

### RT‐qPCR

Total RNA was extracted from gastric biopsies using an RNeasy Mini Kit (Qiagen, Manchester, UK). After DNase treatment using a TURBO DNA‐free Kit, RNA was reverse‐transcribed into cDNA using a High‐Capacity cDNA Reverse Transcription Kit (both Thermo Fisher Scientific). qPCR was performed on a Rotor‐Gene 3000 using QuantiTect Primer Assays for *CD55* (Hs_DAF_1_SG; QT00099190) and *ACTB* (Hs_ACTB_1_SG; QT00095431) (both Qiagen) with Power SYBR Green Master Mix (Thermo Fisher Scientific). Relative *CD55* levels were calculated using the Pfaffl method [32], normalizing against *ACTB*, and as fold‐differences compared with pooled antral biopsies from uninfected patients.

### Histology and immunohistochemistry

Gastric biopsy sections were stained with H&E, blinded, and scored by an expert gastrointestinal (GI) pathologist for inflammation, atrophy, and IM, using the updated Sydney System [33]. This grades the density of neutrophil and mononuclear cell (MNC) infiltration and the degree of gland loss based on a visual analogue scale: none, mild, moderate, or marked. Similarly, IM (presence of goblet cells) in biopsies was scored as none, mild (involving <25% of crypts), moderate (25–50% of crypts), or marked (>50% of crypts). Where goblet cells could not be clearly discerned on H&E sections, Alcian blue/PAS staining was performed for the presence of acidic mucins. Separate sections were stained with toluidine blue for the presence of *H. pylori*. Immunostaining was performed using Novolink Polymer Detection Systems (Leica Biosystems, Milton Keynes, UK). In brief, after antigen retrieval with citrate buffer (pH 6.0) for 20 min, sections were incubated with rabbit anti‐human CD55 mAb (E7G2U; Cell Signaling Technology, London, UK; 1:1,200), rabbit anti‐human CD4 mAb (EPR6855; Abcam, Cambridge, UK; 1:1,000) or rat anti‐human Pax‐5 mAb (1H9; Biolegend, London, UK; 1:100) at 4 °C overnight. For Pax‐5 staining, sections were further incubated with rabbit anti‐rat IgG (Abcam) for 30 min at room temperature. After incubation with Rabbit Novolink polymer (anti‐rabbit Poly‐HRP‐IgG) for 30 min at room temperature, staining was visualised with DAB, followed by haematoxylin counterstain. Mucosal CD55 was assessed independently by two scientists and a histopathologist using light microscopy, blinded to the endoscopy findings, histopathological scores, and RT‐qPCR results. CD55 immunohistochemistry scores were calculated by multiplying staining intensity (0, negative; 1, weak; 2, moderate; 3, strong) by staining distribution (0, no staining; 1, <25%; 2, 26–50%; 3, 51–75%; 4, >75%), and observers’ scores were averaged. Staining on lymphoid aggregates was excluded from the scoring.

### sCD55 and IL‐8 ELISA

sCD55 and IL‐8 were quantified using the Human CD55/DAF ELISA Pair Set (Sino Biological, Beijing, PR China) and IL‐8 Human Uncoated ELISA kit (Thermo Fisher Scientific) respectively, following the manufacturers’ instructions.

### Western blotting

After washing with PBS, cells were solubilised in radioimmunoprecipitation assay buffer containing 0.1 m PMSF. Cellular proteins (10–20 μg) were electrophoresed through 10% SDS‐PAGE gels, transferred to nitrocellulose membranes, and probed with anti‐CD55 (E7G2U, 1:1,000) and anti‐β‐actin (ab8227, 1:1,000, Abcam) antibodies at 4 °C overnight. Membranes were then incubated with alkaline‐phosphatase‐conjugated goat anti‐rabbit secondary Ab (1:10,000, Sigma‐Aldrich) at room temperature for 1 h and visualised using BCIP/NBT substrate (Sigma‐Aldrich).

### Statistical analysis

All statistical analyses were carried out using GraphPad Prism 9 software (GraphPad Software, San Diego, CA, USA). The tests used are indicated in the figure legends. *p* < 0.05 was considered statistically significant.

## Results

### Increased CD55 in gastric mucosal tissue is associated with *H. pylori* infection and IM

**Figure 1:**
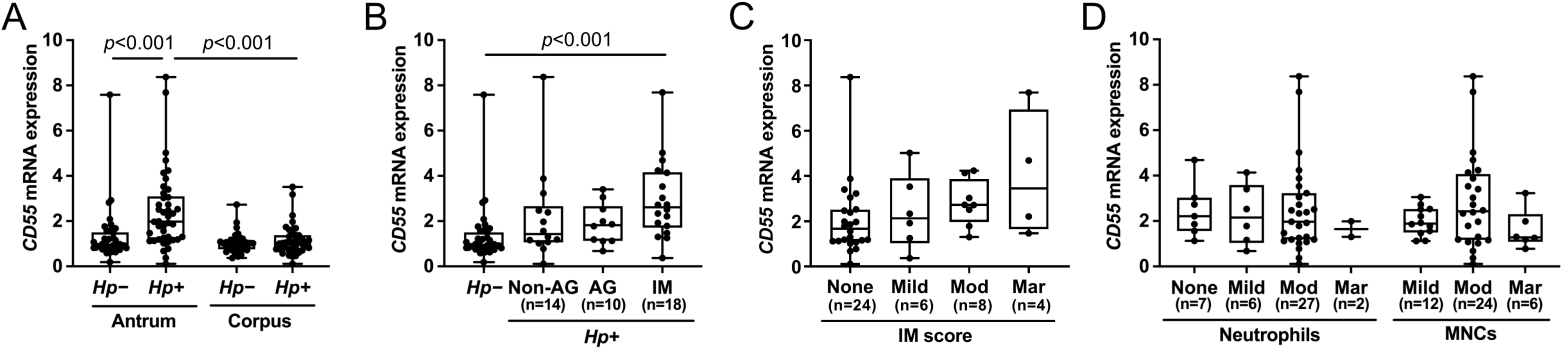
Infection with *Helicobacter pylori* increases *CD55* mRNA in human gastric antrum. The relative levels of *CD55* mRNA in gastric mucosal tissues from 42 infected and 42 uninfected patients were analysed by RT‐qPCR. Data were normalised to *ACTB* mRNA and expressed relative to pooled RNA from antral tissue samples from uninfected patients. Levels were compared between (A) uninfected and infected patients in the antrum and corpus; (B) infected patients with and without atrophy or IM in the antrum; (C) the antrum of infected patients with varying IM score; and (D) varying infiltration scores of neutrophils and MNCs in infected antral samples. Each dot represents an individual patient's data. The horizontal line in each box represents the median value, with the boxes representing the interquartile range. Lines extend from the box to the highest and lowest values. *P* values were calculated using (A) two‐way ANOVA with Tukey's *post hoc* test and (B–D) Kruskal–Wallis with Dunn's *post hoc* test. AG, atrophic gastritis; *Hp*, *H. pylori*; IM, intestinal metaplasia; MNCs, mononuclear cells; Mod, moderate; Mar, marked.

To determine whether *H. pylori* infection was associated with CD55 expression in the gastric mucosa, RT‐qPCR was performed on patient biopsies. In the antrum, *CD55* mRNA was significantly increased in *H. pylori*‐infected compared with uninfected patients (Figure 1A). When these antral data were stratified according to histological characteristics, samples from infected patients displaying IM showed significantly higher *CD55* than uninfected patients (Figure 1B), and expression tended to correlate with IM score (Figure 1C). In contrast, *CD55* mRNA was not associated with atrophy or the degree of inflammation, as determined by neutrophil and MNC infiltrations (Figure 1B,D). *CD55* mRNA in infected and uninfected corpus tissues was similar (Figure 1A), likely due to few samples with IM (6/42), most of which were mild (supplementary material, Figure S1).

**Figure 2:**
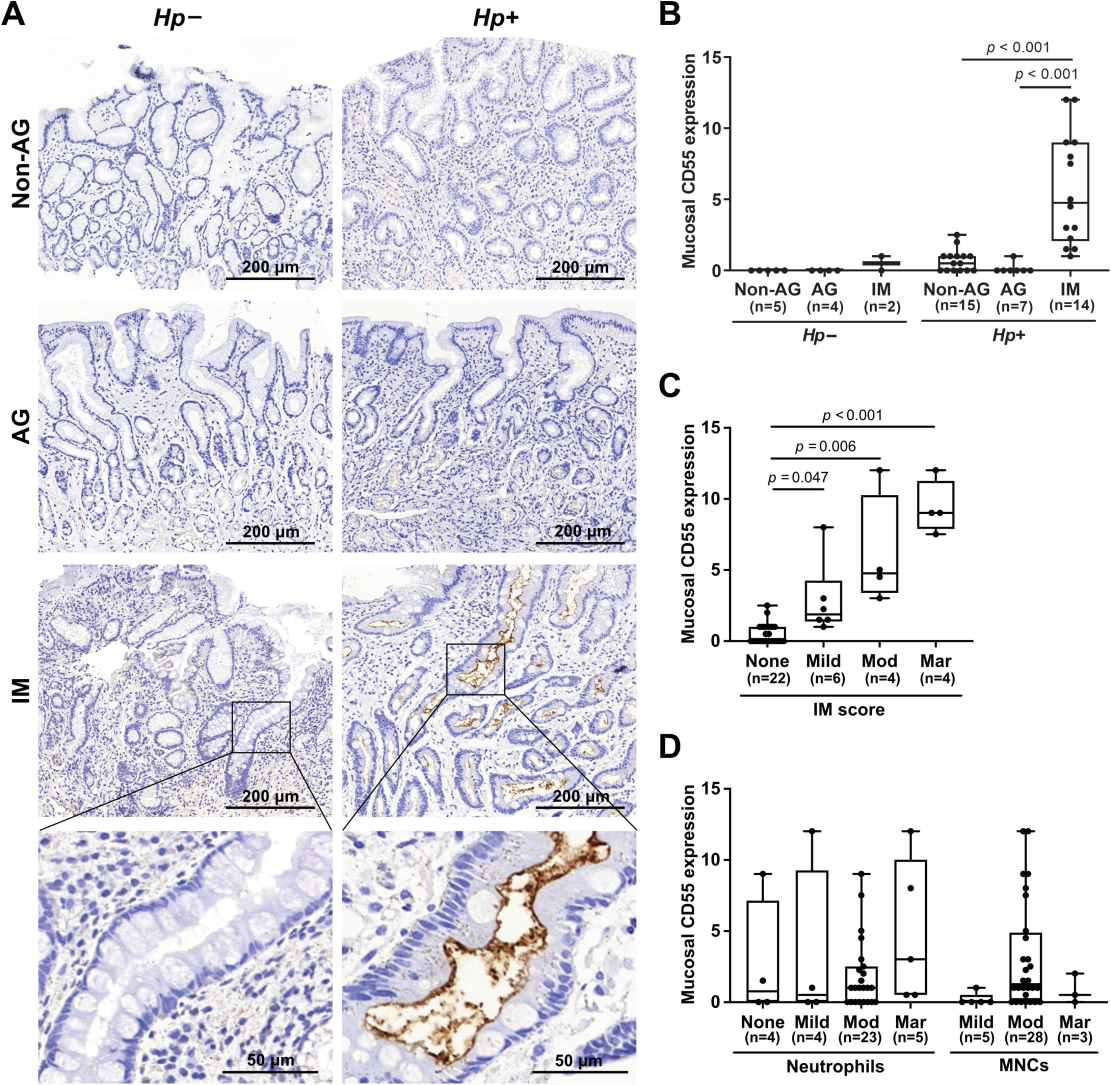
CD55 in *H. pylori*‐infected gastric epithelium is associated with intestinal metaplasia but not inflammation. Gastric antrum biopsy tissues from 36 infected and 11 uninfected patients were stained immunohistochemically for CD55. (A) Representative images of stained sections. (B) Each section was scored semi‐quantitatively based on the distribution and intensity of mucosal staining. (C, D) CD55 IHC scores of infected epithelium samples were stratified by IM score and the amount of neutrophil and MNC infiltration. Each dot represents an individual patient's data. The horizontal line in each box represents the median value, with the boxes representing the interquartile range. Lines extend from the box to the highest and lowest values. *P* values were calculated using (B) two‐way ANOVA with Tukey's *post hoc* test and (C, D) Kruskal–Wallis with Dunn's *post hoc* test. AG, atrophic gastritis; *Hp*, *H. pylori*; IM, intestinal metaplasia; MNCs, mononuclear cells; Mod, moderate; Mar, marked.

For comparison with the RT‐qPCR results, antral tissue sections were immunostained for CD55. The apical surface of epithelium from *H. pylori*‐infected patients stained strongly for CD55, among patients with IM (Figure 2A). When scored on the combined distribution and intensity of staining, mucosal CD55 expression was significantly associated with IM and positively correlated with IM score (Figure 2B,C). Mucosal CD55 was not influenced by neutrophil or MNC infiltration (Figure 2D), although lymphoid aggregates also stained strongly for CD55 (supplementary material, Figure S2A). CD55 staining appeared localised to aggregate centres rather than in peripheral areas and was a very different pattern to CD4, a marker of helper T lymphocytes, indicating that CD4^+^ T lymphocytes were not the source of CD55. The staining of Pax‐5, a pan‐B lymphocyte marker, was more similar to that of CD55, but Pax‐5 staining was also present in the surrounding area where CD55 staining was weak or absent (supplementary material, Figure S2A). Lymphoid aggregates were present in approximately 60% (22/36) of infected antral tissues, compared with 18% (2/11) of uninfected tissues. Mucosal CD55 was more common in infected tissues with lymphoid aggregates than without (Table 2). Nevertheless, aggregate presence had no significant impact on *CD55* mRNA or CD55 protein levels (supplementary material, Figure S2B), suggesting that neither *H. pylori*‐induced lymphoid aggregates nor inflammation directly drives mucosal CD55 expression.

**Table 2:** Presence of gastric lymphoid aggregates and mucosal CD55 expression.

Lymphoid aggregates	*H. pylori‐*negative		*H. pylori‐positive*	
	Positive (*n* = 2)	Negative (*n* = 9)	Positive (*n* = 22)	Negative (*n* = 14)
Mucosal CD55				
Positive, *n* (%)	1 (50.0%)	0 (0.0%)	17 (77.3%)	7 (50.0%)
Negative, *n* (%)	1 (50.0%)	9 (100.0%)	5 (22.7%)	7 (50.0%)

### The virulence factor VacA regulates expression of *CD55*

**Figure 3:**
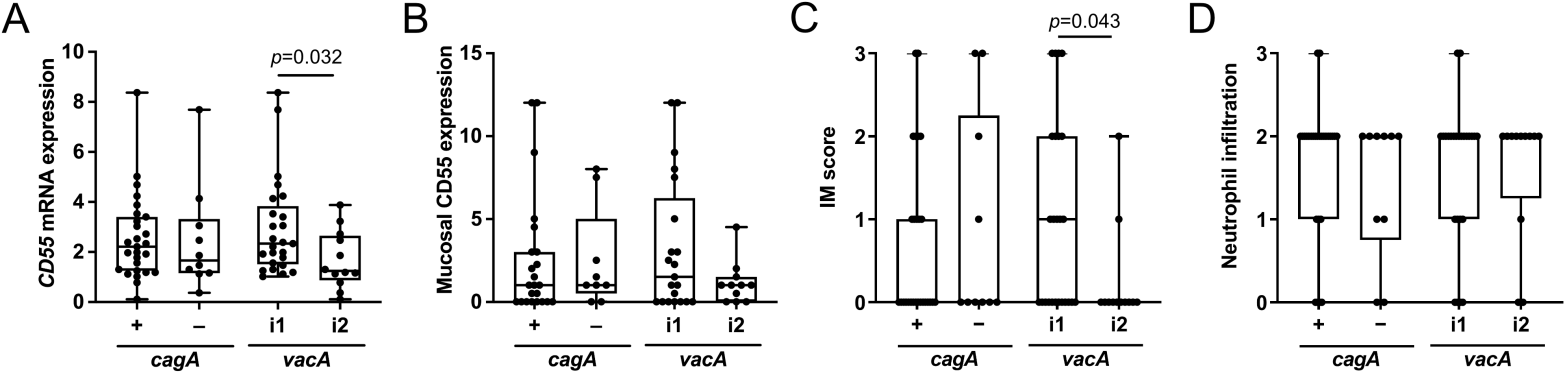
Gastric expression of CD55 and intestinal metaplasia are associated with *H. pylori* strains of *vacA* i1 genotype. (A) *CD55* mRNA; (B) mucosal CD55 IHC scores; and Sydney System scores (0, none; 1, mild; 2, moderate; and 3, marked) for (C) IM and (D) neutrophil infiltration, in the gastric antrum of 37 patients infected with *H. pylori* strains of different virulence genotypes (*cagA* ^+^, *n* = 27; *cagA* ^*−*^, *n* = 10; *vacA* i1, *n* = 25; *vacA* i2, *n* = 12). Each dot represents an individual patient's data. The horizontal line in each box represents the median value, with the boxes representing the interquartile range. Lines extend from the box to the highest and lowest values. *P* values were calculated using a Mann–Whitney *U*‐test. IM, intestinal metaplasia.

Since we found previously that IM is strongly associated with colonisation by *vacA* s1i1‐type strains [20], we hypothesised that VacA plays a role in inducing *CD55*. Stratifying the gastric *CD55* expression data according to *vacA* type and *cagA* status, significantly higher *CD55* mRNA was observed in patients infected with *vacA* i1‐type strains than with i2‐type, whereas no difference was found between those infected with *cagA* ^+^ and *cagA* ^−^ strains (Figure 3A). Patients who had higher CD55 protein tended to be infected with *vacA* i1‐type strains, but this was not statistically significant (Figure 3B). IM Sydney scores were significantly higher in individuals infected with *vacA* i1‐type strains than with i2‐type, but there was no association with *cagA* status (Figure 3C). It should be noted that antral inflammation was not associated with either *vacA* type or *cagA* status (Figure 3D).

**Figure 4:**
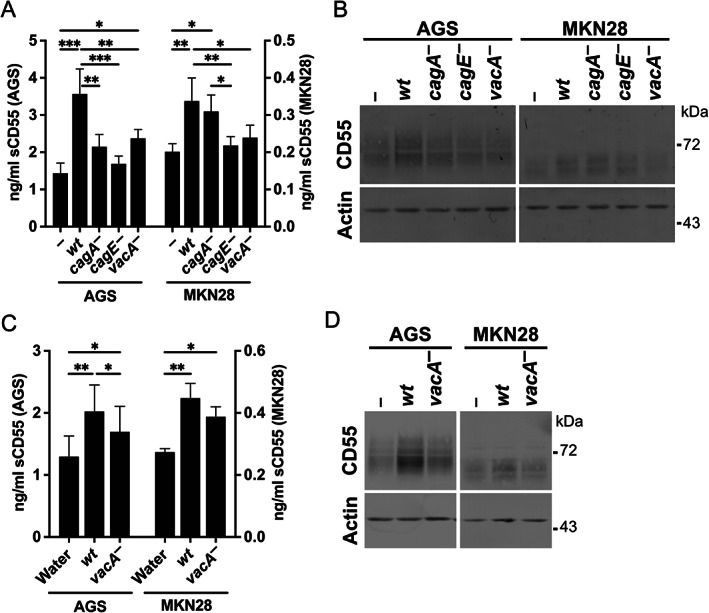
Increased CD55 expression and secretion by *H. pylori* are mediated via *vacA* and *cag*T4SS. (A, B) AGS or MKN28 cells were co‐cultured with *H. pylori* strain 60190 or isogenic mutants for 24 h at an MOI of 20. (C, D) AGS or MKN28 cells were incubated for 24 h in the presence of 100 μg/ml total protein of water extracts of *H. pylori* strain 60190 or isogenic *vacA* ^−^ mutant. (A, C) sCD55 concentrations in culture supernatants were determined by ELISA. Mean ± SEM from three or four independent experiments. *p < 0.05; **p < 0.01; ***p < 0.001 by one‐way ANOVA with Tukey's *post hoc* test. (B, D) Western blot of CD55 in cell lysates. wt, wild type.

To look for causal relationships between VacA and CD55, AGS and MKN28 cells were co‐cultured with *H. pylori*, and cellular expression and secretion of CD55 were analysed. Both cell lines expressed CD55 constitutively, but they differed in expression level and pattern; AGS cells secreted nearly 10‐fold more sCD55 (supplementary material, Figure S3A) and expressed more glycosylated forms of CD55 than MKN28 cells (supplementary material, Figure S3B). The detected bands were confirmed to correspond to CD55 using siRNA knockdown (supplementary material, Figure S6B). Compared with uninfected cells, both sCD55 concentration and CD55 expression were significantly increased in both cell lines by co‐culture with *H. pylori* strain 60190 (*cagA* ^+^, *vacA* s1i1m1) in a dose‐dependent manner (supplementary material, Figure S3). As expected, when AGS and MKN28 cells were co‐cultured with isogenic *cagA*, *cagE*, and *vacA* null mutants of strain 60190, no hummingbird phenotype was induced by *cagA* ^−^ and *cagE* ^−^ mutants and the *vacA* ^−^ mutant did not induce vacuolation (supplementary material, Figure S4). In line with previous reports [26, 27], 60190 *cagE* ^−^, which lacks *cag*T4SS function, failed to induce sCD55 secretion in AGS and MKN28 cells; concentrations were similar to uninfected cells (Figure 4A). Similarly, the *vacA* ^−^ mutant induced lower sCD55 concentrations than wild‐type 60190 for both cell lines. However, while the *cagA* ^−^ mutant induced significantly less sCD55 in AGS cells, it induced similar levels to that of the wild‐type strain in MKN28 cells. Similar trends were observed in CD55 in response to the bacterial mutants (Figure 4B). Additionally, when MKN28 cells were co‐cultured with *cag*T4SS‐inactive SS1 strain mutants expressing different *vacA* alleles, the s1i1 mutant tended to induce more sCD55 and CD55 than the s1i2 or s2i2 mutants, but the differences were not statistically significant (supplementary material, Figure S5). Next, cells were treated with water extracts prepared from wild‐type and *vacA* ^−^ mutant strains. sCD55 concentrations and cellular CD55 were increased in response to both preparations, but the *vacA* ^−^ mutant extract was less effective (Figure 4C,D). Collectively, these data suggest that in addition to the *cag*T4SS, VacA can induce CD55 directly and indirectly.

### *H. pylori* ‐induced IL‐8 response in gastric epithelial cells is partly mediated by CD55

**Figure 5:**
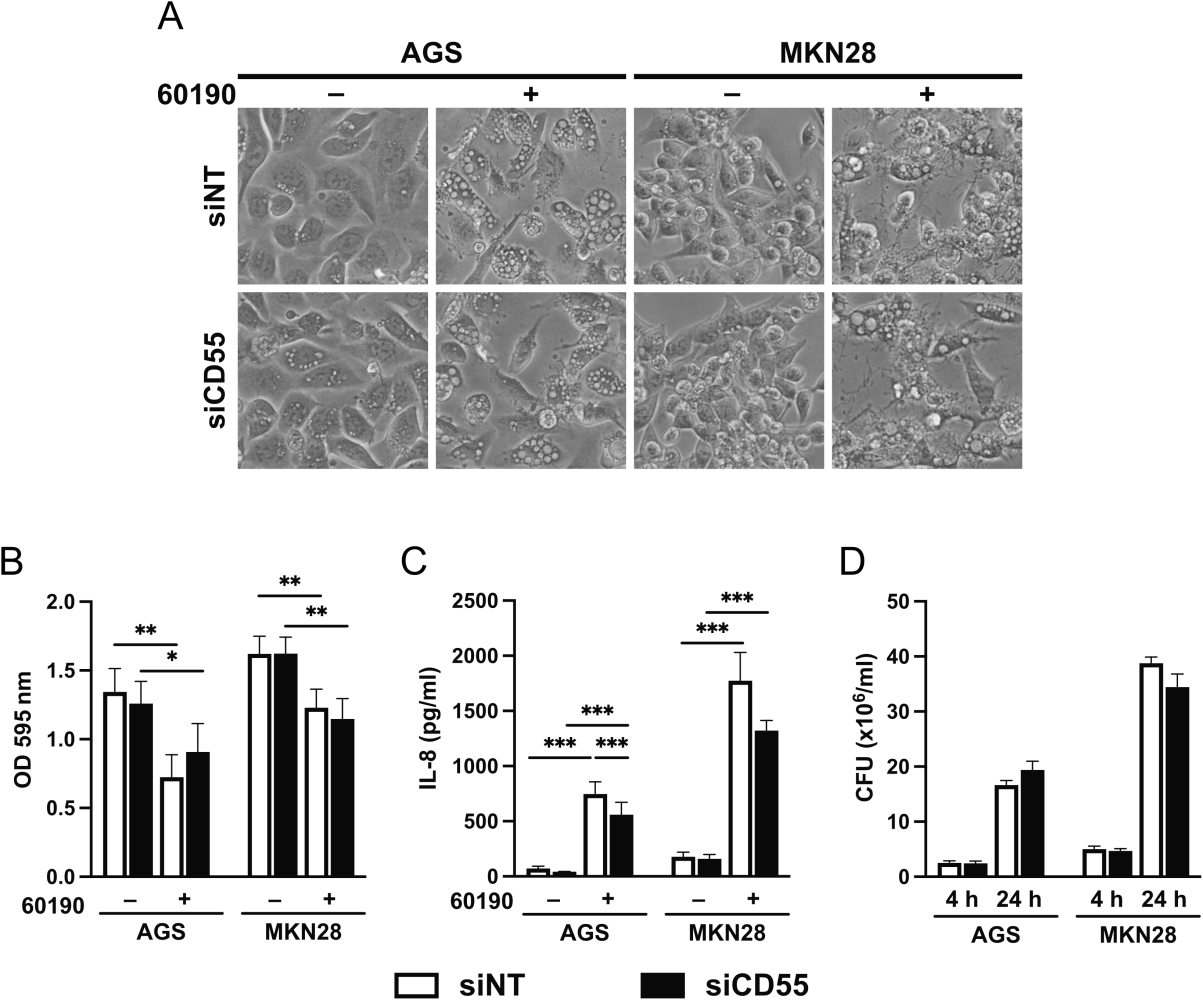
CD55 knockdown reduces IL‐8 production but has no effect on *H. pylori* binding to gastric epithelial cells. AGS or MKN28 cells transfected with *CD55* siRNA (siCD55) or non‐targeting control (siNT) were co‐cultured with *H. pylori* strain 60190. (A) Representative images of cell morphology 24 h post‐infection. (B) Cell viability 24 h post‐infection as measured using an MTT assay. (C) IL‐8 concentrations in cell culture supernatants determined by ELISA 24 h post‐infection. (D) Adherence of *H. pylori* to epithelial cells at 4 and 24 h post‐infection. Mean ± SEM from four independent experiments. *p < 0.05; **p < 0.01; ***p < 0.001 by two‐way ANOVA with Tukey's *post hoc* test.

To investigate whether CD55 has any effects on gastric epithelial cells, endogenous *CD55* mRNA was silenced by siRNA in AGS and MKN28 cells (>80% knockdown measured by RT‐qPCR). While significantly reduced sCD55 and CD55 proteins were seen in knockdown cells (supplementary material, Figure S6), *H. pylori*‐induced cell morphology (Figure 5A) and cell viability (Figure 5B) were both unaffected. Epithelial cells secrete IL‐8 in response to *H. pylori*, which is largely dependent on interactions with the *cag*T4SS [34]. Thus, we hypothesised that IL‐8 induction may be at least partially mediated by CD55. Co‐culture of gastric epithelial cell lines with *H. pylori* significantly increased IL‐8 secretion, as expected. When we compared cells transfected with *CD55* siRNA with non‐targeting controls, *CD55* knockdown resulted in a 25% reduction in average IL‐8 induced by *H. pylori* (Figure 5C). Previous reports suggest that overexpression of CD55 increases attachment of *H. pylori* to cells [26]. To test whether the observed effect on IL‐8 response was due to a difference in bacterial adhesion, we compared *H. pylori* binding to cells transfected with either *CD55* or non‐targeting siRNAs. Similar levels of bacteria attached to both cell transfectants after 4 and 24 h co‐culture (Figure 5D). Overall, these results suggest that CD55 is partly responsible for the IL‐8 response of gastric epithelial cells to *H. pylori*, which is a novel finding.

### Plasma sCD55 concentrations do not correlate with gastric CD55 levels

**Figure 6:**
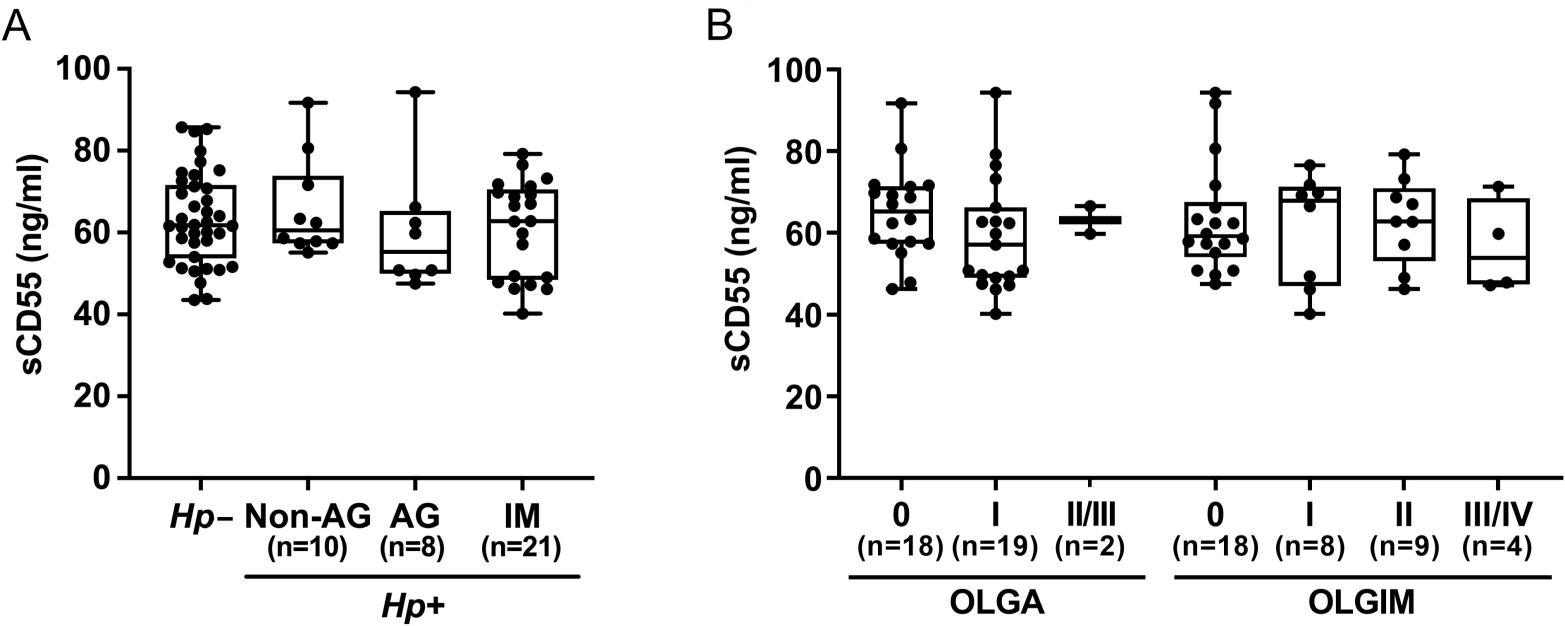
Comparison of plasma sCD55 levels in patients with gastric atrophy and intestinal metaplasia. (A) sCD55 concentrations in plasma samples from 39 infected and 38 uninfected patients were quantified by ELISA. (B) sCD55 concentrations in infected patients were stratified by Operative Link on Gastritis (OLGA) and Operative Link on Gastritis based on IM (OLGIM) stage. Each dot represents an individual patient's data. The horizontal line in each box represents the median value, with the boxes representing the interquartile range. Lines extend from the box to the highest and lowest values. *Hp*, *H. pylori*; AG, atrophic gastritis; IM, intestinal metaplasia.

The major form of CD55 is membrane‐bound, although sCD55 generated by alternative splicing or by MMP‐7 has been detected in human plasma [21, 29]. As an association was found between gastric CD55 and IM, we hypothesised that plasma sCD55 could be a marker for the presence of early pre‐malignant changes. Using ELISA, sCD55 was measured in plasma samples from the same patients. sCD55 concentrations were similar between uninfected and infected patients with non‐AG, AG, and IM, and did not correlate with gastric *CD55* mRNA (Figure 6A). sCD55 concentrations in infected patients were further stratified by Operative Link on Gastritis (OLGA) [35, 36] and Operative Link on Gastritis based on IM (OLGIM) stage, which combines the antrum and corpus Sydney score for AG and IM, respectively. However, there was no significant difference in sCD55 concentrations between OLGA or OLGIM stages (Figure 6B).

## Discussion

In agreement with other studies [24, 25], antral gastric biopsies from *H. pylori*‐infected patients displaying IM showed *CD55* mRNA and protein significantly higher than those from uninfected patients. Additionally, CD55 on the apical surface of epithelium was significantly associated with IM score, although weak CD55 staining was occasionally observed in the absence of IM. Neither the presence of atrophy nor the degree of inflammation was associated with *CD55* mRNA or protein levels. In contrast, Sasaki *et al* [28] previously reported a strong correlation between gastric CD55 expression and the degree of neutrophil and mononuclear cell infiltration. This discrepancy is most likely due to not stratifying their data by *H. pylori* status as the majority of specimens with moderate to marked infiltration were from infected patients, whereas those with no or mild infiltration were from uninfected patients. Interestingly, their subsequent publication showed that gastric CD55 decreased 1 month after *H. pylori* eradication, suggesting that CD55 is regulated by *H. pylori* infection [37]. Importantly, their staining seems not to be limited to metaplastic mucosa, which is different from our findings and those of others [24, 25]. Further studies are required to investigate whether *H. pylori* eradication decreases CD55 on metaplastic mucosa.

In addition to metaplastic mucosa, we found that lymphoid aggregates stained strongly for CD55 (supplementary material, Figure S2A). Since the role of CD55 in T‐cell immunity has been demonstrated [22, 23], we were surprised that the staining patterns of CD55 and CD4 were very different. Indeed, CD55 localised to germinal centres, the sites of B‐cell expansion and selection, and the presence of B cells in the germinal centres was confirmed by Pax‐5 staining. This finding is supported by recent data showing CD55 localisation in tonsil germinal centres and that its expression on B‐cell surfaces regulates their complement‐dependent phagocytosis for maintaining homeostasis [38]. Furthermore, our data showed that CD55 staining in lymphoid aggregates was independent of *H. pylori* status and that their presence was not associated with gastric mucosal *CD55* mRNA levels. Therefore, CD55 on gastric immune cells is likely to be independent of *H. pylori*‐induced metaplastic changes in the stomach.

Our data showed that both high CD55 and IM were more common in individuals infected with *vacA* i1‐type strains than with i2‐type, suggesting a link between CD55, IM, and *vacA* i1 genotype. Causal relationships between VacA and CD55 were confirmed in subsequent *in vitro* studies infecting AGS and MKN28 gastric epithelial cells with *vacA* mutant strains. In agreement with others [27], we found that in addition to VacA, *H. pylori* utilises the *cag*T4SS to induce CD55. Although the involvement of CagA was also observed in AGS (but not MKN28) cells, we found no association of CD55 or IM score with *cagA* status in gastric biopsies. Additionally, almost half of *vacA* i2 strains were *cagA* ^+^, and IM and CD55 were present in three of four patients infected with *cagA* ^−^ *vacA* i1 strains. Therefore, the observed associations are likely to be CagA‐independent.

Results from our siRNA experiments suggest that CD55 partially regulates the IL‐8 response of epithelial cells to *H. pylori*. The mechanism is unlikely to involve modulation of bacterial adhesion or cytotoxicity because bacterial attachment and cell viability were similar in CD55 knockdown and control cells. Additionally, gastric CD55 was enhanced in areas of IM which generally occur at a later stage of infection, suggesting that CD55 is not a main adhesin for *H. pylori*. We and others have previously shown that MAPK and/or NF‐κB pathways regulate *H. pylori‐*induced IL‐8 expression [34, 39, 40], and chronic activation of these pathways is well known to play a role in carcinogenesis [41]. The work by O'Brien *et al* demonstrated that *H. pylori* induces CD55 via activation of the p38 MAPK pathway independently of NF‐κB signalling in gastric epithelial cells [27], suggesting that CD55 signalling could be an accessory factor in MAPK activation, and a strengthening of this response via CD55 signalling is likely to cause more damaging effects including progression to (and increased extent of) IM. Both the *cag*T4SS and VacA have been shown to activate the p38 MAPK pathway, but our previous data suggest that IL‐8 expression by gastric epithelial cell lines is *cag*T4SS‐dependent but independent of VacA [34, 39, 42, 43], which may be the reason why CD55 knockdown only partially reduced the IL‐8 response. Whether the regulation of IL‐8 by CD55 is relevant to the development of IM remains to be investigated. Interestingly, p38 MAPK mediates intestinal epithelial cell differentiation via activation of CDX2 [44], an intestinal epithelial‐specific transcription factor found in gastric IM and intestinal‐type adenocarcinoma [45]. CDX2 may play an important role in IM development since transgenic mice expressing CDX2 in the stomach display features of human IM, such as the presence of goblet cells and expression of intestine‐specific genes [46, 47]. Future research needs to explore potential relationships between CDX2 and CD55.

In our cohort, no association was found between plasma sCD55 and IM, AG or *H. pylori* status. CD55 is expressed on the cell surface of nearly all peripheral blood cells and is present on a wide range of tissues [48]. Since we detected high concentrations of sCD55 in plasma, secretion of sCD55 from gastric tissue may have been masked by secretion from other tissues. However, these results need to be validated in larger cohorts before a firm conclusion can be drawn. An IgM antibody against a tumour‐specific isoform of CD55 has been found to promote gastric tumour regression by inducing apoptosis, without causing severe side effects [49]; therefore, CD55 inhibition, or its use as a ligand to induce apoptosis in lesions, may help to inhibit the progression of IM. However, IM does not always lead to GC and there remains a debate on whether IM is the direct precursor to GC [50].

In conclusion, our data strengthen the evidence for an association between CD55 and IM in the *H. pylori*‐infected human gastric mucosa and provide a new insight into the role of VacA in the development of GC. There is a great deal of interest in preventing the progression of IM to GC. Further longitudinal studies of the link between CD55 and progression of IM could demonstrate the potential of CD55 as a therapeutic target, as previously explored in colorectal cancer [51].

## Author contributions statement

KK, KR, JCA and IS conceived and designed the study. KK, AMZ, DPL, JLR, JCA and JT carried out the experiments. KK, AMZ, DPL and KR analysed and interpreted the data. All the authors were involved in writing the manuscript and approved the final version.

## Supporting information


**Figure S1.** *CD55* mRNA in human gastric corpus
**Figure S2.** CD55 is present in *H. pylori*‐induced lymphoid aggregates
**Figure S3.** *H. pylori* increases expression and secretion of CD55 in gastric epithelial cells
**Figure S4.** Characteristic morphological changes in gastric epithelial cells infected with *H. pylori* mutants
**Figure S5.** CD55 expression and secretion in gastric epithelial cells after infection with *H. pylori* strains of different *vacA* genotypes
**Figure S6.** CD55 knockdown in gastric epithelial cells

## References

[1] Sung H , Ferlay J , Siegel RL , *et al*. Global cancer statistics 2020: GLOBOCAN estimates of incidence and mortality worldwide for 36 cancers in 185 countries. CA Cancer J Clin 2021; 71: 209–249. 33538338 10.3322/caac.21660

[2] Robinson K , Atherton JC . The spectrum of *Helicobacter*‐mediated diseases. Annu Rev Pathol 2021; 16: 123–144. 33197219 10.1146/annurev-pathol-032520-024949

[3] Correa P , Haenszel W , Cuello C , *et al*. A model for gastric cancer epidemiology. Lancet 1975; 2: 58–60. 49653 10.1016/s0140-6736(75)90498-5

[4] Shah SC , Gawron AJ , Mustafa RA , *et al*. Histologic subtyping of gastric intestinal metaplasia: overview and considerations for clinical practice. Gastroenterology 2020; 158: 745–750. 31887261 10.1053/j.gastro.2019.12.004 PMC7302270

[5] Pimentel‐Nunes P , Libânio D , Marcos‐Pinto R , *et al*. Management of epithelial precancerous conditions and lesions in the stomach (MAPS II): European Society of Gastrointestinal Endoscopy (ESGE), European Helicobacter and Microbiota Study Group (EHMSG), European Society of Pathology (ESP), and Sociedade Portuguesa de Endoscopia Digestiva (SPED) guideline update 2019. Endoscopy 2019; 51: 365–388. 30841008 10.1055/a-0859-1883

[6] González CA , Sanz‐Anquela JM , Gisbert JP , *et al*. Utility of subtyping intestinal metaplasia as marker of gastric cancer risk. A review of the evidence. Int J Cancer 2013; 133: 1023–1032. 23280711 10.1002/ijc.28003 PMC3732516

[7] Banks M , Graham D , Jansen M , *et al*. British Society of Gastroenterology guidelines on the diagnosis and management of patients at risk of gastric adenocarcinoma. Gut 2019; 68: 1545–1575. 31278206 10.1136/gutjnl-2018-318126 PMC6709778

[8] Chen HN , Wang Z , Li X , *et al*. *Helicobacter pylori* eradication cannot reduce the risk of gastric cancer in patients with intestinal metaplasia and dysplasia: evidence from a meta‐analysis. Gastric Cancer 2016; 19: 166–175. 25609452 10.1007/s10120-015-0462-7

[9] Rokkas T , Rokka A , Portincasa P . A systematic review and meta‐analysis of the role of *Helicobacter pylori* eradication in preventing gastric cancer. Ann Gastroenterol 2017; 30: 414–423. 28655977 10.20524/aog.2017.0144 PMC5479993

[10] Lin AS , Dooyema SDR , Frick‐Cheng AE , *et al*. Bacterial energetic requirements for *Helicobacter pylori* Cag type IV secretion system‐dependent alterations in gastric epithelial cells. Infect Immun 2020; 88: e00790‐19. 31712269 10.1128/IAI.00790-19 PMC6977121

[11] Argent RH , Kidd M , Owen RJ , *et al*. Determinants and consequences of different levels of CagA phosphorylation for clinical isolates of *Helicobacter pylori* . Gastroenterology 2004; 127: 514–523. 15300584 10.1053/j.gastro.2004.06.006

[12] Olbermann P , Josenhans C , Moodley Y , *et al*. A global overview of the genetic and functional diversity in the *Helicobacter pylori cag* pathogenicity island. PLoS Genet 2010; 6: e1001069. 20808891 10.1371/journal.pgen.1001069 PMC2924317

[13] Miernyk KM , Bruden D , Rudolph KM , *et al*. Presence of *cag*PAI genes and characterization of *vacA* s, i and m regions in *Helicobacter pylori* isolated from Alaskans and their association with clinical pathologies. J Med Microbiol 2020; 69: 218–227. 32011229 10.1099/jmm.0.001123 PMC10874806

[14] Plummer M , van Doorn LJ , Franceschi S , *et al*. *Helicobacter pylori* cytotoxin‐associated genotype and gastric precancerous lesions. J Natl Cancer Inst 2007; 99: 1328–1334. 17728213 10.1093/jnci/djm120

[15] Basso D , Zambon CF , Letley DP , *et al*. Clinical relevance of *Helicobacter pylori cagA* and *vacA* gene polymorphisms. Gastroenterology 2008; 135: 91–99. 18474244 10.1053/j.gastro.2008.03.041

[16] Matos JI , de Sousa HA , Marcos‐Pinto R , *et al*. *Helicobacter pylori CagA* and *VacA* genotypes and gastric phenotype: a meta‐analysis. Eur J Gastroenterol Hepatol 2013; 25: 1431–1441. 23929249 10.1097/MEG.0b013e328364b53e

[17] Chauhan N , Tay ACY , Marshall BJ , *et al*. *Helicobacter pylori* VacA, a distinct toxin exerts diverse functionalities in numerous cells: an overview. Helicobacter 2019; 24: e12544. 30324717 10.1111/hel.12544

[18] Rhead JL , Letley DP , Mohammadi M , *et al*. A new *Helicobacter pylori* vacuolating cytotoxin determinant, the intermediate region, is associated with gastric cancer. Gastroenterology 2007; 133: 926–936. 17854597 10.1053/j.gastro.2007.06.056

[19] Atherton JC , Cao P , Peek RM Jr , *et al*. Mosaicism in vacuolating cytotoxin alleles of *Helicobacter pylori*. Association of specific *vacA* types with cytotoxin production and peptic ulceration. J Biol Chem 1995; 270: 17771–17777. 7629077 10.1074/jbc.270.30.17771

[20] Winter JA , Letley DP , Cook KW , *et al*. A role for the vacuolating cytotoxin, VacA, in colonization and *Helicobacter pylori*‐induced metaplasia in the stomach. J Infect Dis 2014; 210: 954–963. 24625807 10.1093/infdis/jiu154 PMC4136800

[21] Dho SH , Lim JC , Kim LK . Beyond the role of CD55 as a complement component. Immune Netw 2018; 18: e11. 29503741 10.4110/in.2018.18.e11 PMC5833118

[22] Capasso M , Durrant LG , Stacey M , *et al*. Costimulation via CD55 on human CD4^+^ T cells mediated by CD97. J Immunol 2006; 177: 1070–1077. 16818763 10.4049/jimmunol.177.2.1070

[23] Liu J , Miwa T , Hilliard B , *et al*. The complement inhibitory protein DAF (CD55) suppresses T cell immunity *in vivo* . J Exp Med 2005; 201: 567–577. 15710649 10.1084/jem.20040863 PMC2213052

[24] Berstad AE , Brandtzaeg P . Expression of cell membrane complement regulatory glycoproteins along the normal and diseased human gastrointestinal tract. Gut 1998; 42: 522–529. 9616315 10.1136/gut.42.4.522 PMC1727075

[25] Kiso T , Mizuno M , Nasu J , *et al*. Enhanced expression of decay‐accelerating factor and CD59/homologous restriction factor 20 in intestinal metaplasia, gastric adenomas and intestinal‐type gastric carcinomas but not in diffuse‐type carcinomas. Histopathology 2002; 40: 339–347. 11943018 10.1046/j.1365-2559.2002.01350.x

[26] O'Brien DP , Israel DA , Krishna U , *et al*. The role of decay‐accelerating factor as a receptor for *Helicobacter pylori* and a mediator of gastric inflammation. J Biol Chem 2006; 281: 13317–13323. 16543227 10.1074/jbc.M601805200

[27] O'Brien DP , Romero‐Gallo J , Schneider BG , *et al*. Regulation of the *Helicobacter pylori* cellular receptor decay‐accelerating factor. J Biol Chem 2008; 283: 23922–23930. 18579524 10.1074/jbc.M801144200 PMC2527108

[28] Sasaki M , Joh T , Tada T , *et al*. Altered expression of membrane inhibitors of complement in human gastric epithelium during Helicobacter‐associated gastritis. Histopathology 1998; 33: 554–560. 9870151 10.1046/j.1365-2559.1998.00539.x

[29] Medof ME , Walter EI , Rutgers JL , *et al*. Identification of the complement decay‐accelerating factor (DAF) on epithelium and glandular cells and in body fluids. J Exp Med 1987; 165: 848–864. 2434600 10.1084/jem.165.3.848 PMC2188295

[30] Boughan PK , Argent RH , Body‐Malapel M , *et al*. Nucleotide‐binding oligomerization domain‐1 and epidermal growth factor receptor: critical regulators of beta‐defensins during *Helicobacter pylori* infection. J Biol Chem 2006; 281: 11637–11648. 16513653 10.1074/jbc.M510275200

[31] Letley DP , Rhead JL , Twells RJ , *et al*. Determinants of non‐toxicity in the gastric pathogen *Helicobacter pylori* . J Biol Chem 2003; 278: 26734–26741. 12738773 10.1074/jbc.M304071200

[32] Pfaffl MW . A new mathematical model for relative quantification in real‐time RT‐PCR. Nucleic Acids Res 2001; 29: e45. 11328886 10.1093/nar/29.9.e45 PMC55695

[33] Dixon MF , Genta RM , Yardley JH , *et al*. Classification and grading of gastritis. The updated Sydney System. International Workshop on the Histopathology of Gastritis, Houston 1994. Am J Surg Pathol 1996; 20: 1161–1181. 8827022 10.1097/00000478-199610000-00001

[34] Cook KW , Letley DP , Ingram RJ , *et al*. CCL20/CCR6‐mediated migration of regulatory T cells to the *Helicobacter pylori*‐infected human gastric mucosa. Gut 2014; 63: 1550–1559. 24436142 10.1136/gutjnl-2013-306253 PMC4173663

[35] Rugge M , Genta RM , OLGA Group . Staging gastritis: an international proposal. Gastroenterology 2005; 129: 1807–1808. 16285989 10.1053/j.gastro.2005.09.056

[36] Capelle LG , de Vries AC , Haringsma J , *et al*. The staging of gastritis with the OLGA system by using intestinal metaplasia as an accurate alternative for atrophic gastritis. Gastrointest Endosc 2010; 71: 1150–1158. 20381801 10.1016/j.gie.2009.12.029

[37] Joh T , Sasaki M , Kataoka H , *et al*. *Helicobacter pylori* eradication decreases the expression of glycosylphosphatidylinositol‐anchored complement regulators, decay‐accelerating factor and homologous restriction factor 20, in human gastric epithelium. J Gastroenterol Hepatol 2005; 20: 1344–1351. 16105119 10.1111/j.1440-1746.2005.03876.x

[38] Dernstedt A , Leidig J , Holm A , *et al*. Regulation of decay accelerating factor primes human germinal center B cells for phagocytosis. Front Immunol 2020; 11: 599647. 33469456 10.3389/fimmu.2020.599647 PMC7813799

[39] Allison CC , Kufer TA , Kremmer E , *et al*. *Helicobacter pylori* induces MAPK phosphorylation and AP‐1 activation via a NOD1‐dependent mechanism. J Immunol 2009; 183: 8099–8109. 20007577 10.4049/jimmunol.0900664

[40] Tran CT , Garcia M , Garnier M , *et al*. Inflammatory signaling pathways induced by *Helicobacter pylori* in primary human gastric epithelial cells. Innate Immun 2017; 23: 165–174. 27913793 10.1177/1753425916681077

[41] Asokan S , Bandapalli OR . CXCL8 signaling in the tumor microenvironment. Adv Exp Med Biol 2021; 1302: 25–39. 34286439 10.1007/978-3-030-62658-7_3

[42] Ki MR , Lee HR , Goo MJ , *et al*. Differential regulation of ERK1/2 and p38 MAP kinases in VacA‐induced apoptosis of gastric epithelial cells. Am J Physiol Gastrointest Liver Physiol 2008; 294: G635–G647. 18096609 10.1152/ajpgi.00281.2007

[43] Nakayama M , Kimura M , Wada A , *et al*. *Helicobacter pylori* VacA activates the p38/activating transcription factor 2‐mediated signal pathway in AZ‐521 cells. J Biol Chem 2004; 279: 7024–7028. 14630932 10.1074/jbc.M308898200

[44] Houde M , Laprise P , Jean D , *et al*. Intestinal epithelial cell differentiation involves activation of p38 mitogen‐activated protein kinase that regulates the homeobox transcription factor CDX2. J Biol Chem 2001; 276: 21885–21894. 11283019 10.1074/jbc.M100236200

[45] Bai YQ , Yamamoto H , Akiyama Y , *et al*. Ectopic expression of homeodomain protein CDX2 in intestinal metaplasia and carcinomas of the stomach. Cancer Lett 2002; 176: 47–55. 11790453 10.1016/s0304-3835(01)00753-4

[46] Mutoh H , Hakamata Y , Sato K , *et al*. Conversion of gastric mucosa to intestinal metaplasia in Cdx2‐expressing transgenic mice. Biochem Biophys Res Commun 2002; 294: 470–479. 12051735 10.1016/S0006-291X(02)00480-1

[47] Silberg DG , Sullivan J , Kang E , *et al*. Cdx2 ectopic expression induces gastric intestinal metaplasia in transgenic mice. Gastroenterology 2002; 122: 689–696. 11875002 10.1053/gast.2002.31902

[48] Lublin DM , Atkinson JP . Decay‐accelerating factor: biochemistry, molecular biology, and function. Annu Rev Immunol 1989; 7: 35–58. 2469439 10.1146/annurev.iy.07.040189.000343

[49] Hensel F , Timmermann W , von Rahden BH , *et al*. Ten‐year follow‐up of a prospective trial for the targeted therapy of gastric cancer with the human monoclonal antibody PAT‐SC1. Oncol Rep 2014; 31: 1059–1066. 24452482 10.3892/or.2014.2987 PMC3926647

[50] Graham DY , Zou WY . Guilt by association: intestinal metaplasia does not progress to gastric cancer. Curr Opin Gastroenterol 2018; 34: 458–464. 30138135 10.1097/MOG.0000000000000472 PMC6913177

[51] Dho SH , Cho EH , Lee JY , *et al*. A novel therapeutic anti‐CD55 monoclonal antibody inhibits the proliferation and metastasis of colorectal cancer cells. Oncol Rep 2019; 42: 2686–2693. 31578581 10.3892/or.2019.7337

