# Supplementary material for: The active form of Helicobacter pylori vacuolating cytotoxin induces decay‐accelerating factor CD55 in association with intestinal metaplasia in the human gastric mucosa
Source: J Pathol. 2022 Aug 18;258(2):199–209. doi: 10.1002/path.5990 (PMC9543990; doi:10.1002/path.5990)
Supplement: Supplementary file 1 — Figure S1. CD55 mRNA in human gastric corpus Figure S2. CD55 is present in H. pylori‐induced lymphoid aggregates Figure S3. H. pylori increases expression and secretion of CD55 in gastric epithelial cells Figure S4. Characteristic morphological changes in gastric epithelial cells infected with H. pylori mutants Figure S5. CD55 expression and secretion in gastric epithelial cells after infection with H. pylori strains of different vacA genotypes Figure S6. CD55 knockdown in gastric epithelial cells [file PATH-258-199-s001.docx]

**The active form of *Helicobacter pylori* vacuolating cytotoxin induces decay-accelerating factor CD55 in association with intestinal metaplasia in the human gastric mucosa**

K Kaneko *et al. J Pathol* <https://doi.org/10.1002/path.5990>

**Supplementary Figures S1–S6**

**

**

**Figure S1.** ***CD55* mRNA in human gastric corpus.** *CD55* mRNA in gastric corpus tissues from 39 infected and 42 uninfected patients was analysed by real-time RT-qPCR. Data were normalised to *ACTB* and expressed relative to antral tissue samples from uninfected patients. (A) *CD55* mRNA in the corpus of uninfected and infected patients with and without atrophy or IM. (B) *CD55* mRNA in the corpus of infected patients with varying IM score. Each dot represents an individual patient’s data. The horizontal line in each box represents the median value, with the boxes representing the interquartile range. Lines extend from the box to the highest and lowest values. AG, atrophic gastritis; *Hp*, *H. pylori*; IM, intestinal metaplasia; Mod, moderate.

**
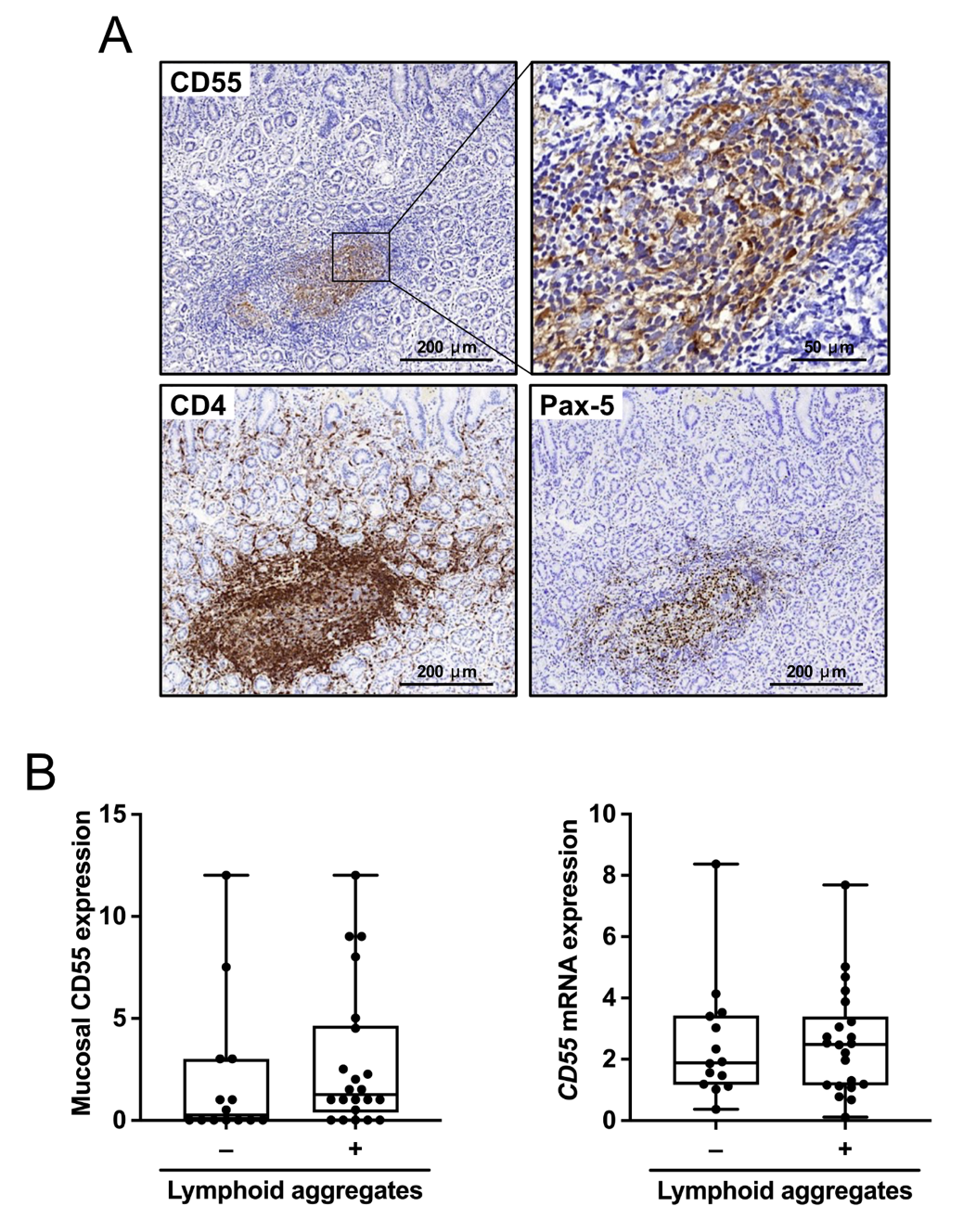
**

**Figure S2. CD55 is present in *H. pylori*-induced lymphoid aggregates.** Gastric antrum biopsy tissues from infected and uninfected patients were stained immunohistochemically for CD55, CD4 (helper T lymphocyte marker), and Pax-5 (pan-B lymphocyte marker. (A) Representative images of stained sections. (B) CD55 immunohistochemistry scores and mRNA levels in infected antrum with (*n*= 22) and without (*n* = 14) lymphoid aggregates. Each dot represents an individual patient’s data. The horizontal line in each box represents the median value, with the boxes representing the interquartile range. Lines extend from the box to the highest and lowest values.

A

B

72

43

**CD55**

**Actin**

kDa

**50**

**AGS**

**0**

**10**

**20**


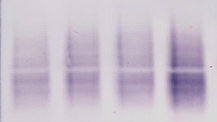

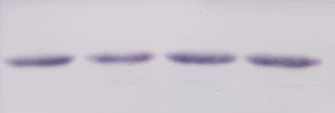


**MOI**

**50**

**MKN28**

**0**

**10**

**20**


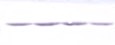

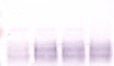


**Figure S3. *H. pylori* increases expression and secretion of CD55 in gastric epithelial cells.** AGS or MKN28 cells were co-cultured with *H. pylori* strain 60190 for 24 h. (A) sCD55 concentrations in culture supernatants were determined by ELISA. Mean ± SEM from five independent experiments. **p* < 0.05; ***p* < 0.01; ****p* < 0.001 by one-way ANOVA with Tukey’s *post hoc* test. (B) Western blot of CD55 in cell lysates. MOI, multiplicity of infection.

**
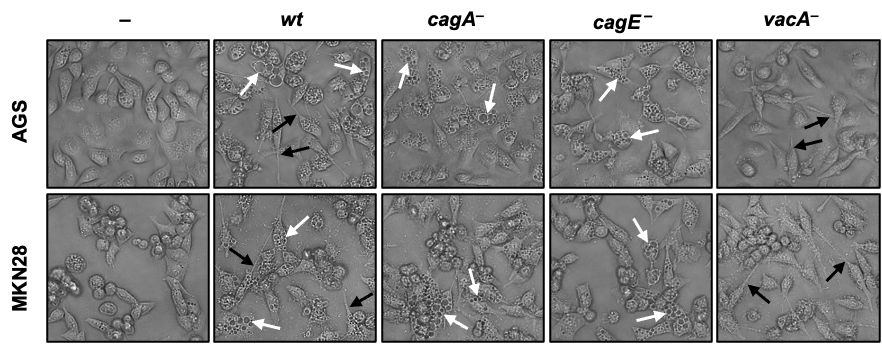
**

**Figure S4. Characteristic morphological changes in gastric epithelial cells infected with *H. pylori* mutants.** AGS or MKN28 cells were co-cultured with *H. pylori* strain 60190 or isogenic *cagA*^–^, *cagE*^–^ or *vacA*^–^ mutants for 24 h at an MOI of 20. Magnification: 40× objective. wt, wild type. White arrows indicate cytoplasmic vacuolation; black arrows indicate hummingbird phenotype (cellular protrusions).

**

**

**Figure S5. CD55 expression and secretion in gastric epithelial cells after infection with *H. pylori* strains of different *vacA* genotypes.** MKN28 cells were co-cultured with *H. pylori* strain SS1 expressing s1i1, s1i2, s2i2 or null *vacA* for 24 h at an MOI of 100. (A) sCD55 concentrations in culture supernatants were determined by ELISA. Mean ± SEM from three independent experiments. **p* < 0.05; ***p* < 0.01; ****p* < 0.001 by one-way ANOVA with Tukey’s *post hoc* test. (B) Western blot analysis of CD55 in cell lysates and VacA in culture supernatant.

**

**

**Figure S6. CD55 knockdown in gastric epithelial cells.** AGS or MKN28 cells transfected with *CD55* siRNA (siCD55) or non-targeting control (siNT) were co-cultured with *H. pylori* strain 60190 at an MOI of 20. (A) sCD55 concentrations in culture supernatants 24 h post-infection were determined by ELISA. Mean ±  SEM from three or four independent experiments. **p* < 0.05 by two-way ANOVA with Tukey’s *post hoc* test. (B) Western blot of CD55 in cell lysates 4 and 24 h post-infection.
